# HLA Polymorphism and Susceptibility to End-Stage Renal Disease in Cantonese Patients Awaiting Kidney Transplantation

**DOI:** 10.1371/journal.pone.0090869

**Published:** 2014-03-06

**Authors:** Qiong Cao, Di Xie, Jiangmei Liu, Hongyan Zou, Yinze Zhang, Hong Zhang, Zhimei Zhang, Hao Xue, Jiyuan Zhou, Pingyan Chen

**Affiliations:** 1 Division of Tissue Typing Center, Nanfang Hospital, Southern Medical University, Guangdong, Guangzhou, China; 2 Division of Nephrology, Nanfang Hospital, Southern Medical University, Guangdong, Guangzhou, China; 3 Department of Biostatistics, School of Public Health and Tropical Medicine, Southern Medical University, Guangdong, Guangzhou, China; 4 HLA High-Resolution Confirmatory Typing Laboratory, Shenzhen Blood Center, Shenzhen, China; University of Cape Town, South Africa

## Abstract

**Background:**

End-Stage Renal Disease (ESRD) is a worldwide public health problem. Currently, many genome-wide association studies have suggested a potential association between human leukocyte antigen (HLA) and ESRD by uncovering a causal relationship between HLA and glomerulonephritis. However, previous studies, which investigated the HLA polymorphism and its association with ESRD, were performed with the modest data sets and thus might be limited. On the other hand, few researches were conducted to tackle the Chinese population with ESRD. Therefore, this study aims to detect the susceptibilities of HLA polymorphism to ESRD within the Cantonese community, a representative southern population of China.

**Methods:**

From the same region, 4541 ESRD patients who were waiting for kidney transplantation and 3744 healthy volunteer bone marrow donors (controls) were randomly chosen for this study. Polymerase chain reaction-sequence specific primer method was used to analyze the HLA polymorphisms (including HLA-A, HLA-B and HLA-DRB1 loci) in both ESRD patients and controls. The frequencies of alleles at these loci and haplotypes were compared between ESRD patients and controls.

**Results:**

A total of 88 distinct HLA alleles and 1361 HLA A-B-DRB1 haplotypes were detected. The frequencies of five alleles, HLA-A*24, HLA-B*55, HLA-B*54, HLA-B*40(60), HLA-DRB1*04, and one haplotype (HLA-A*11-B*27-DRB1*04) in ESRD patients are significantly higher than those in the controls, respectively.

**Conclusions:**

Five HLA alleles and one haplotype at the HLA-A, HLA-B and HLA-DRB1 loci appear to be associated with ESRD within the Cantonese population.

## Introduction

With a high incidence, end-stage renal disease (ESRD) becomes a worldwide public health problem [1]. ESRD is a condition where a patient is permanently dependent on renal replacement in order to avoid life-threatening uremia. The incidence of ESRD has been increasing in Europe and United States over the past decade, with doubling the number of patients [2]. In China, there is a rising incidence of ESRD, too. The number of the registered ESRD patients who were treated by hemodialysis was 41,755 in 1999, and this number was even more than 120,000 in 2008 [3]. The annual incidence of ESRD that needed hemodialysis therapy was estimated to be as high as 36.1 per million population (pmp) in China [4].

Many patients with chronic kidney disease (CKD) can progress to ESRD despite receiving intensive therapy, and the rate of progression varies from person to person. Exploring those specific genetic-variants in ESRD patients can benefit the development of novel strategies to detect and prevent ESRD at the early stage. Unlike developed countries, in which the major causes of ESRD are diabetes mellitus and hypertension, the leading cause of ESRD in China remains glomerulonephritis, which accounts for 49.9% of total kidney diseases [4]–[6]. Recently, many genome-wide association studies have detected a strong association between the human leukocyte antigen (HLA) and glomerulonephritis [7]–[10], indicating a potential association between HLA and ESRD. HLA is located at chromosome 6p21.31 [11]. People with certain HLA types are more likely to developing autoimmune diseases such as type I diabetes. Moreover, HLA has a gene-dense region, and its mutation is also linked to autoimmune diseases [11]. Therefore, HLA plays a critical role in the immune responses, which are crucial in ESRD processing. Exploring the specific genetic-variants of HLA in ESRD patients may benefit the development of novel strategies to detect and prevent ESRD at the early stage. Thus, this study expands our knowledge by analyzing the association between HLA polymorphism and ERSD, and detects the mechanisms underlying initiation and progression of renal failure.

Currently, there have been several studies to indicate the association between HLA alleles/haplotypes and ESRD [12], [13]. However, these findings were based on the modest data sets and thus might be limited. On the other hand, little is known about the association between HLA polymorphism and ESRD within the Chinese population, specifically in the Cantonese patients. In order to enrich the knowledge of HLA polymorphism in the Cantonese population and detect its susceptibilities to ESRD, the frequencies of alleles at the HLA-A, -B and -DRB1 loci and haplotypes in both ESRD patients who are waiting for kidney transplantation and healthy volunteer bone marrow donors (controls) from the same region of Southern China were investigated.

## Methods

### Patients and control donors

4541 Cantonese patients in 1996–2010, who were diagnosed with ESRD and waiting for a cadaveric kidney, were selected from the Transplantation Center of Nanfang Hospital for this study. As the largest transplantation center of Southern China, Transplantation Center of Nanfang Hospital holds the database of tissue typing, and it also provides HLA-A, -B and -DRB1genotype data of the patients. The medical records of the patients such as age, gender, and primary cause of ESRD were extracted from Nanfang Hospital. All the data were collected by a research nurse, who is a non-investigator in this study. In order to preserve patients' privacy, all data are anonymous.

Based on the database of HLA High-Resolution Confirmatory Typing Laboratory of Shenzhen Blood Center, a total 3744 unrelated, healthy, and volunteer bone marrow donors were selected as controls from the same Southern Chinese population. The age, gender, HLA-A, -B and -DRB1 genotype data of the donors were acquired from a registry system database. The laboratory of Shenzhen Blood Center is one of seven HLA high-resolution confirmatory typing laboratories of the China Marrow Donor Program, which is a non-profit sub-organization of the Red Cross Society of China. All the data of the controls were hosted by Shenzhen Blood Center. All the identifying information of volunteers was removed in the final dataset for analysis in order to preserve patients' privacy.

The entire study protocol was approved by the Nanfang Ethics Committee, and the whole research process was supervised by Nanfang Ethics Committee.

### DNA extraction

Whole blood samples were collected from the participants and stored at −20°C until DNA extraction. Genomic DNA was extracted from whole blood samples containing ethylene diamine tetraacetic acid (EDTA) by QIAamp DNA blood Mini Kit (QIAGEN GmbH, Hilden, Germany), which can yield good quality high molecular weight DNA suitable for analysis [14].

### Polymerase chain reaction-sequence specific primer based typing at HLA-A, -B and–DRB1 loci

The genotyping at the HLA-A, -B, and -DRB1 loci was performed by the Tissue Typing Center affiliated to the Transplantation Center of Nanfang Hospital. The Tissue Typing Center uses a standardized set of HLA typing program, provided by National Center for Clinical Laboratory of China. Polymerase chain reaction (PCR) amplification was performed using a GeneAmp PCR system 9700 (Applied Biosystems, Foster city CA, USA). According to the manufacturer's instructions, sequence specific primer (SSP) analysis was performed with a mixture of nucleotides and dNTPs by HLA-ABDR GeneType analysis kit (Biotest AG, Dreieich, Germany). All amplifications were performed in a thermocycler using the following conditions: initial denaturation 94°C for 2 minutes, denaturation 94°C for 10 seconds, annealing and extension 65°C for 1 minute, 10 cycles, followed by 94°C for 10 seconds, 61°C for 50 seconds, 72°C of 30 seconds for 20 cycles, and stored at 4°C. Primer set amplified single amplicons as demonstrated by agarose gel electrophoresis. The PCR products were pre-stained with SYBR Green I (0.5 µl/100 ml gel), loaded in agarose gels, and then electrophoresed for 10 minutes at 10V/cm in 0.5×TBE (Tris-Boric acid-EDTA) buffer. The agarose gels were examined under UV illumination and documented by photography. The exact HLA type was investigated by Biotest HLA-SSP Typing software 1.1.

### Statistical methods

Data are described with arithmetic mean, standard deviation, median, range and absolute number of the subjects. The frequencies of the alleles at the HLA-A, -B and -DRB1 loci were estimated using SAS9.17®. The frequencies of the HLA-A-B-DRB1 haplotypes were calculated by the expectation maximization (EM) algorithm using Arlequin software 3.5 (Switzerland). The exact test was used to evaluate the assumption of Hardy-Weinberg equilibrium (HWE) as described by Guo and Thomson [15]. The linkage disequilibrium coefficient between any two alleles at these loci is measured by Lewontin D' [16]. The frequencies of the HLA-A, -B, -DRB1 alleles and HLA-A-B-DRB1 haplotypes were compared between ESRD patients and controls by Fisher's exact test or Pearson chi-square test with the Bonferroni correction for multiple testing [17]. A 5% significance level was considered sufficient to reject the null hypothesis.

## Results

### Characteristics of patients and control donors

A total of 4541 Cantonese ESRD patients were selected from the database of the tissue typing center affiliated with the Transplantation Center of Nanfang Hospital, including 2754 (60.65%) males. The median age of patients was 40 (mean±sd: 40±12, min-max: 7–81). Among those patients, 1975 (43.5%) patients have glomerulonephritis, which is the most common cause of ESRD. A total of 3744 healthy volunteer bone marrow donors were included in this study. All donors were unrelated, and from the same southern Chinese population, including 1994 (53.26%) males. The median age was 31.0 (mean±sd: 32±8, min-max: 18–55).

### Hardy-Weinberg equilibrium tests at HLA-A,-B and -DRB1 loci

In 4541 ESRD patients, there were 21 HLA-A, 47 HLA-B and 14 HLA-DRB1 alleles to occur. In 3744 control donors, there were 17 HLA-A, 41 HLA-B and 16 HLA-DRB1 alleles to occur. In both groups, a total of 88 distinct HLA alleles occurred including 21 HLA-A, 51 HLA-B and 16 HLA-DRB1 alleles (see Table 1). The most frequent alleles which occurred at the HLA-A, -B and–DRB1 loci of all subjects were consistent with a previous study conducted within the Cantonese population [18]. The HWE tests at the HLA-A, -B and -DRB1 loci showed the violation of HWE in both the ESRD patients and the controls (P<0.05).

**Table 1:** Numbers of HLA-A, -B and -DRB1 alleles in ESRD patients and controls.

Locus	No. of alleles		
	ESRD patients	Controls	Both groups
HLA-A	21	17	21
HLA-B	47	41	51
HLA-DRB1	14	16	16

### Allele frequency at HLA-A, -B and -DRB1 loci in kidney transplant recipients and control donors

The frequencies of alleles at the HLA-A, -B and -DRB1 loci obtained by DNA typing are summarized in Table 2. In the most frequent alleles (top 20% of all the allele frequencies) which occurred at the HLA-A (n = 4), -B (n = 10) and -DRB1 (n = 4) loci in the ESRD patients or in the controls, the frequencies of HLA-A*24, HLA-B*55, HLA-B*54, HLA-B*40(60), HLA-DRB1*04 were significantly higher in ESRD patients than those in controls, respectively.

**Table 2:** Allele frequencies at HLA-A, -B and -DRB1 loci in ESRD patients and controls (ordered by statistical significance for each locus).

Allele	ESRD patients a (n = 4541*2)	Controls a (n = 3744*2)	P b	Adjusted P c
**HLA-A (%)**				
*24	17.45	15.37	0.0003	0.0055
**HLA-B (%)**				
*55	3.50	2.50	0.0002	0.0071
*54	3.70	2.67	0.0002	0.0076
*40(60)	16.26	14.37	0.0008	0.0297
**HLA-DRB1 (%)**				
*04	14.21	10.24	<0.0001	<0.0001

a Listed are only the most frequent (top 20%) alleles for each HLA locus in the ESRD patients and controls, respectively.

b Using Fisher exact test.

c P values were adjusted by Bonferroni method. Multiplicative factor was used for each allele.

### HLA-A-B-DRB1 haplotype frequencies and association analysis

Using the EM algorithm, a total of 1361 HLA A-B-DRB1 haplotypes were detected, where there were 974 haplotypes identified in 4541 ESRD patients, and 887 haplotypes identified in 3744 control donors, respectively. Moreover, the frequencies of the three most common haplotypes HLA-A*02-B*46-DRB1*09, HLA-A*33-B*58-DRB1*03(17), and HLA-A*11-B*15(75)-DRB1*12 were 4.42%, 3.97%, and 3.18% in ESRD patients, respectively. Additionally, the above three haplotypes were also the most common haplotypes in the controls, with the frequencies of 4.97%, 5.01%, and 3.61%, respectively. These results were consistent with a previous study conducted within the Cantonese population [18].

For the most frequent HLA-A-B-DRB1 haplotypes (top 5% of all the haplotypes), the haplotype distribution in ESRD patients (n = 50) was significantly different from that in the controls (n = 21), as shown in Table 3. HLA-A*11-B*27-DRB1*04 is one of the common haplotypes in ESRD patients, with the frequency being 0.426%. However, the frequency of the same haplotype was 0.086% in the controls, which was much smaller than that in ESRD patients (*P*<0.05). This result indicated that HLA-A*11-B*27-DRB1*04 haplotype appears to be associated with ESRD within the Cantonese population.

**Table 3:** Frequency of the susceptible three-locus HLA haplotypes in ESRD patients and controls (ordered by statistical significance for susceptible haplotypes).

HLA haplotype a	ESRD patients (n = 4541*2)	Controls (n = 3744*2)	P b	Adjusted P c
**Susceptible HLA A-B-DRB1 haplotype (%)**				
A*11-B*27-DRB1*04	0.426	0.086	<0.0001	0.0036
A*24-B*40(60)-DRB1*08	0.605	0.216	0.0001	0.0514
A*02-B*40(60)-DRB1*11	0.850	0.397	0.0003	0.1450
A*24-B*40(60)-DRB1*04	0.847	0.480	0.0043	1.0000
A*11-B*55-DRB1*04	0.557	0.279	0.0062	1.0000
A*11-B*40(60)-DRB1*04	1.615	1.127	0.0076	1.0000
A*24-B*40(60)-DRB1*15	0.645	0.366	0.0120	1.0000
A*24-B*46-DRB1*09	0.810	0.530	0.0300	1.0000
A*24-B*13-DRB1*15	0.793	0.521	0.0352	1.0000
A*02-B*40(60)-DRB1*12	0.588	0.359	0.0426	1.0000

a Listed are only the top 5% of all the HLA-A-B-DRB1 haplotypes with significant uncorrected P-value.

b Using Fisher exact test.

c P values were adjusted by Bonferroni method. Multiplicative factor was used for each haplotype.

## Discussion

Our study has academic significance. Due to the importance of the immune response in the processing of ESRD, genes located on HLA potentially contribute to the ESRD processing. The identification and analysis of HLA polymorphism are important not only for the study of the ESRD susceptibility, but also for the tissue transplantation in ESRD patients. A key step for tissue transplantation is histocompatibility testing, which is crucial in the selection of tissue receptors and tissue donors. Our study provides useful information for the selection of donor kidneys in Cantonese ESRD patients, who are waiting for kidney transplantation. In these patients, grafted kidney may survive better through selecting donor kidneys without susceptible haplotypes of ESRD, although the efficacy of such an approach for improving the prognosis of kidney transplantation need be supported by further investigations.

Our present study was distinct because we found several HLA alleles/haplotypes which appear to be associated with ESRD. Specifically, significantly higher frequencies of five alleles (HLA-A*24, HLA-B*55, HLA-B*54, HLA-B*40(60) and HLA-DRB1*04) and one haplotype (HLA-A*11-B*27-DRB1*04) (see Tables 2 and 3) in Cantonese ESRD patients were observed, respectively. To further explore specific HLA alleles/haplotypes for specific renal diseases, a subgroup analysis was performed based on definite pathologic diagnosis for the case of enough sample size. Among 4541 ESRD patients, 399 patients received renal biopsy previously and were diagnosed definitely as glomerulonephritis (n = 265), hypertensive nephropathy (n = 32), diabetic nephropathy (n = 39), chronic interstitial nephritis (n = 36), hereditary or other kidney disorders (n = 27), separately. Note that the analysis results based on data sets with small sample size may not be so reliable and thus we only conducted the subgroup analysis based on 265 patients with glomerulonephritis. The frequencies of three alleles (HLA-A*11, HLA-B*58 and HLA-DRB1*04) and one haplotype (HLA-A*02-B*40(61)-DRB1*04) in the 265 patients were significantly higher than those in the controls, respectively (Table 4).

**Table 4:** Allele and haplotype frequencies at HLA-A, -B and -DRB1 loci in ESRD patients with definite pathologic diagnosis of glomerulonephritis and controls.

Allele/Haplotype	ESRD patients a (n = 265*2)	Controls a (n = 3744*2)	P b	Adjusted P c
HLA-A*11 (%)	38.68	32.39	0.0035	0.0104
HLA-B *58 (%)	5.84	9.51	0.0050	0.0241
HLA-DRB1 *04 (%)	14.15	10.24	0.0065	0.0196
HLA-A*02-B*40(61)-DRB1*04(%)	1.077	0.126	0.0002	0.0100

a Listed are only the most frequent (top 20%) alleles for each HLA locus in the ESRD patients and controls, respectively and a HLA-A-B-DRB1 haplotype with significant uncorrected P-value.

b Using Fisher exact test.

c P values were adjusted by Bonferroni method. Multiplicative factor was used for each allele or haplotype.

Note that the polymorphism of HLA-DRB1 is considered as a susceptible genetic marker for several autoimmune conditions and diseases, such as type I diabetes and dilated cardiomyopathy [19], [20]. As such, among the above identified HLA alleles and haplotype, it is worthy to pay close attention to HLA-DRB1*04 which has the frequency of 14.21% in ESRD patients. In our study, either in the analysis for pooled ESRD patients or in subgroup analysis, the frequency of HLA-DRB1*04 in ESRD patients was significantly higher than that in the controls and HLA-DRB1*04 was also included in the HLA-A-B-DRB1 haplotypes which distributed significantly differently between ESRD patients and controls (in the pooled ESRD: HLA-A*11-B*27-DRB1*04; in the glomerulonephritis subgroup: HLA-A*02-B*40(61)-DRB1*04). Another interesting issue is HLA-B*40, with a frequency of 16.26% in ESRD patients, which is identified as a susceptible allele for IgA nephropathy in Han Chinese through a genome-wide association study [8]. HLA associated IgA nephropathy has a high prevalence in Asia, and it is the primary reason for glomerulonephritis among individuals undergoing renal biopsy [21], [22]. Approximately 15–40% patients with HLA associated IgA nephropathy can progress to ESRD within 20 years [23], [24]. In brief, compared to previous studies, our study detected a similar relationship between HLA polymorphism and ESRD incidence. However, discrepancies occurred between our results and other results. For example, compared to the controls, the frequencies of HLA-B*78 and -DRB1*11 significantly increased in 105 Brazilian patients with ESRD; while the frequency of HLA-B*14 was significantly lower in them [12]. Moreover, a study of 1620 IgA nephropathy ESRD patients from Eurotranplant found that the frequencies of HLA-B35 and DR5 (by HLA antigen typing) were significantly increased in these patients, and HLA-A2-B5-DR5 was identified as a susceptible haplotype in ESRD patients [13]. Although further studies are needed, HLA alleles and haplotype which were found to be associated with ESRD in our study may contribute to be susceptible markers for ESRD patients in the Chinese people, especially in the Cantonese people.

CKD is becoming the major pathogenic hypothesis for kidney damage with abnormalities in both humoral and cellular responses, and CKD can progress to the ESRD over a period of time. Logically, the processing of ESRD should be: HLA polymorphism and susceptibility→a disease (one by one) → CKD →ESRD. Regardless of the primary underlying disease, chronically injured kidneys are histomorphologically characterized by tubulointerstitial fibrosis which is considered the common pathway of chronic progressive kidney disease. Recent studies provided the evidence that genetic polymorphism and epigenetic variations determine the individual susceptibility of patients to develop rapid progressive kidney disease [25]. In this study, we focused on the final outcomes of ESRD in order to avoid unexplained causes contributed by many known or unknown diseases. From the results, HLA polymorphism and their susceptibility to ESRD are an indicator rather than the direct causes of ESRD. Glomerulonephritis remained the leading cause of ESRD in 2008, although the contribution of diabetes and hypertension to the ESRD slightly increased according to the national survey [4]. In spite of the continuous change in the patient population with time, analysis done in our study was still stylish because the characteristics of ESRD patients are not substantially changed over years [4]. The number of patients having definite causes for ESRD confirmed by previous renal biopsy was too small to conduct disease specific analysis. In spite of this limitation, our results still provided valuable information. Furthermore, little is known about the working mechanism of HLA genes, and further studies are required to develop the methods for predicting and preventing ESRD at early stages.

This study has a number of statistical strengths. First, this analysis was based on the Cantonese population, and reduced the effect of geographic and population diversity on the results. Second, the present study was conducted in a very large population, and only the most frequent (top 20%) HLA alleles and the most frequent (top 5%) HLA haplotypes were chosen for analysis. With those strict requirements, this study can generate sufficiently statistical power to detect a slight effect, and can avoid generating a fluctuated risk estimate [26]. In addition, only the *P* values of less than 0.05 (after the Bonferroni correction for multiple comparisons) were considered statistically significant; therefore, the efficacy of test is kept.

This study also had a few limitations in its design. Controls were healthy volunteer bone marrow donors, but we could not entirely rule out the possibility of the incidence of ESRD in the future. However, the prevalence of ESRD in Cantonese people is low (about 0.1%) [3]; therefore, the selection bias for future incidence of ESRD is negligible. In addition, the China Marrow Donor Program only collected the age, gender, and HLA genotype data of donors. It is hard to compare other important ESRD risk factors such as hypertension, diabetes mellitus, albuminuria, dyslipidemia, hyperuricemia, and smoking to those of ESRD patients. Also, our study was not as systematic as genome-wide association studies, and further studies should analyze the association of other HLA antigens and other non-HLA antigens in ESRD patients. More studies by HLA typing are required to confirm the current findings in other independent individuals. Another limitation is the EM algorithm in our study. EM algorithm was used to estimate the frequencies of HLA A-B-DRB1 haplotypes, and this approach is usually used for diploid genotype data under the condition of HWE in the pooled data of the patients and controls. However, our results revealed that the frequency distribution of HLA alleles was inconsistent with the HWE in both ESRD patients and controls. The accuracy of the frequency estimates derived from the EM algorithm may be queried in our study. However, Fallin et al [27] suggested that the frequency estimates in individual haplotypes *via* the EM algorithm deviate their true values in 5% range for large samples (>100) in unphased diploid genotype data, even in the worst cases. Although there may be a problem for detecting very rare haplotypes among the sampled individuals (frequency in the sample <0.1%), the likelihood that such extremely rare haplotypes contribute appreciably to the risk of disease among the affected individuals in the samples may be very low. Schmidt [28] indicated that the violation of HWE could be ignored if the sample size is large enough. Therefore, EM algorithm can be used to analyze unphased diploid genotype data for determining the disease-predisposing haplotypes in this study.

In summary, this study detected the association between HLA polymorphism and ESRD and its susceptibilities for ESRD within the Cantonese population. Five susceptible alleles and one susceptible haplotype were detected in Cantonese ESRD patients awaiting kidney transplantation. These HLA alleles and haplotype might serve as susceptible genetic marker for ESRD within the Cantonese population. Results from our study should be confirmed in further investigations.
